# Recombinant polypeptide of *Mycobacterium leprae* as a potential tool for serological detection of leprosy

**DOI:** 10.1186/s13568-019-0928-9

**Published:** 2019-12-17

**Authors:** Marcelo dos Santos Barbosa, Iara Beatriz Andrade de Sousa, Simone Simionatto, Sibele Borsuk, Silvana Beutinger Marchioro

**Affiliations:** 1 Faculty of Health Sciences, Federal University of Grande Dourados, Dourados, MS Brazil; 2 Faculty of Biological and Environmental Sciences, Federal University of Grande Dourados, Dourados, MS Brazil; 3 Center of Technological Development, Biotechnology, Federal University of Pelotas, Pelotas, RS Brazil; 4 Immunology Laboratory, Health Science Institute, Federal University of Bahia, Salvador, BA Brazil

**Keywords:** *Mycobacterium leprae*, Recombinant polypeptide, Diagnosis, Leprosy

## Abstract

Current prevention methods for the transmission of *Mycobacterium leprae*, the causative agent of leprosy, are inadequate as suggested by the rate of new leprosy cases reported. Simple large-scale detection methods for *M. leprae* infection are crucial for early detection of leprosy and disease control. The present study investigates the production and seroreactivity of a recombinant polypeptide composed of various *M. leprae* protein epitopes. The structural and physicochemical parameters of this construction were assessed using in silico tools. Parameters like subcellular localization, presence of signal peptide, primary, secondary, and tertiary structures, and 3D model were ascertained using several bioinformatics tools. The resultant purified recombinant polypeptide, designated rMLP15, is composed of 15 peptides from six selected *M. leprae* proteins (ML1358, ML2055, ML0885, ML1811, ML1812, and ML1214) that induce T cell reactivity in leprosy patients from different hyperendemic regions. Using rMLP15 as the antigen, sera from 24 positive patients and 14 healthy controls were evaluated for reactivity via ELISA. ELISA-rMLP15 was able to diagnose 79.17% of leprosy patients with a specificity of 92.86%. rMLP15 was also able to detect the multibacillary and paucibacillary patients in the same proportions, a desirable addition in the leprosy diagnosis. These results summarily indicate the utility of the recombinant protein rMLP15 in the diagnosis of leprosy and the future development of a viable screening test.

## Introduction

The chronic infectious disease leprosy is caused by the intracellular, acid-fast bacillus known as *Mycobacterium leprae* (Araújo 2003). *M. leprae* may cause dermatological and neurological granulomatous lesions on the skin that may lead to varying levels of numbness and incapacitation (Porto et al. 2015). Despite declining numbers of global leprosy cases, the disease is still endemic to many countries, with Brazil, in particular, ranking the second highest in the number of new cases reported (22,940 in 2017 alone) (Vieira et al. 2018). The World Health Organization (WHO) has delineated objectives to stop the transmission of new leprosy cases between 2016 and 2020. Among them, the development of new diagnostic tools is emphasized to be of utmost importance (WHO 2016). Additionally, the WHO proposes a standardized screening and treatment protocol by introducing an operational classification of multibacillary (MB) leprosy upon a positive smear test, regardless of the number of lesions (Reibel et al. 2015).

Well-trained clinicians able to identify clinical signs and symptoms in patients are crucial for an accurate diagnosis of leprosy (Richardus et al. 2017). Delayed diagnosis occurs frequently though, owing to few available clinical experts in the field (Corstjens et al. 2019), and increases the risk of severe disabilities (van Hooij et al. 2019). Other diagnostic methods like bacilloscopy and histopathology also lack adequate sensitivity and rely on well-trained technicians as well (Cheng et al. 2019). Molecular diagnostic methods like PCR and qPCR are difficult and expensive to perform in the field, despite having high levels of sensitivity (Martinez et al. 2014; Cheng et al. 2019). Although serological tests based on *M. leprae* antigens are available, they lack adequate sensitivity and are only for supporting clinical diagnosis (Kim et al. 2013). Although primarily used for detecting MB patients, the phenolic glycolipid I (PGL-I) (Roche et al. 1999) and the Leprosy IDRI Diagnostic-1 (LID-I) tests stand out (Duthie et al. 2007; Hungria et al. 2012). Also of significance is the NDO-LID^®^ test, a rapid serological, lateral flow test designed with two proteins, ND-O (a synthetic PGL-I mimetic) and LID-I (a fusion protein of ML0405 and ML2331) (Reece et al. 2006; Hungria et al. 2017; van Hooij et al. 2018).

A number of *M. leprae* proteins and subsequently, tests based on these proteins, have been developed since elucidation of its genomic sequence (Cole et al. 2001) for serological diagnosis of leprosy (Meeker et al. 1986; Duthie et al. 2007; Hungria et al. 2017). These tests could only detect lepromatous and symptomatic cases, but not paucibacillary (PB) cases (Kumar et al. 2014; Duthie et al. 2014; Bahmanyar et al. 2016). The spectrum of outcomes following *M. leprae* infection is determined by host factors (van Hooij et al. 2019) ranging from anti-inflammatory T helper-2 (Th2)-mediated immunity against high bacterial loads and antibodies against *M. leprae* antigens in MB leprosy to strong pro-inflammatory T helper-1 (Th1) and T helper-17 (Th17)-mediated immunity characteristic of PB leprosy (Saini et al. 2013). The human leukocyte antigen (HLA) alleles are also hypothesized to influence host immune responses against *M. leprae* infection (de Souza-Santana et al. 2015). Thus, a reliable diagnostic test for leprosy should be able to capture the different clinical outcomes of *M. leprae* infection, including both cellular and humoral markers (van Hooij et al. 2019).

In a study by Bobosha et al. (2012), epitopes were identified and synthesized from a virulent group of *M. leprae* proteins with predicted promiscuous binding affinities to HLA class I or II alleles. Immunogenicity was tested using peripheral blood mononuclear cells (PBMCs) or whole blood isolated from patients and healthy endemic controls (HCs) from Brazil, Ethiopia, and Nepal. T-cell reactivity was induced in some hyperendemic patients without inducing cross-reactivity with other *Mycobacterium* species. In light of these results, we propose that unique candidate peptides of *M. leprae* could act as more precise diagnostic targets to measure, alongside the cellular and humoral immune responses. Our hypothesis that the inclusion of epitopes from high T-cell reactive proteins of *M. leprae* to the protein might lead to a better antibody response due to T-cell dependent B-cell activation.

Thus, the current study aimed to generate a single recombinant polypeptide composed of epitopes from high T-cell reactive proteins of *M. leprae* (Bobosha et al. 2012) and validate its seroreactivity in leprosy patients. This is based on previous reports to produce a synthetic protein that combines highly reactive segments of *M. leprae* antigens within a single product.

## Materials and methods

### DNA sequence construction of recombinant polypeptide MLP15

High T-cell reactive epitopes of 15 peptides from six different *M. leprae* proteins studied previously (Bobosha et al. 2012) were combined into a recombinant polypeptide designated rMLP15. Multiple epitopes showing HLA I/II reactivity were also placed at random intervals in the polypeptide. Selected epitopes were separated by 3-glycine linker residues and the Vector-NTI Express v1.1.1 program was used for designing a codon-optimized DNA sequence (for *Escherichia coli*, Epoch Life Science, Houston, TX, USA) of the polypeptide rMLP15 (GenBank accession number MN178257). NCBI’s Blastp (Protein BLAST, Altschul et al. 1990) was then used to analyze and exclude cross-reactive protein fragments of genus *Mycobacterium* (taxid: 81858) and *Homo sapiens* (taxid: 9606).

### In silico analyses

The predictions of physicochemical properties were performed using ProtParam tool (Gasteiger et al. 2005) from ExPASy, which provided molecular weight, theoretical isoelectric point (pI), amino acid composition, extinction coefficient, estimated half-life, instability, and aliphatic indexes. The RaptorX software and web server were used for the estimation of primary, secondary, tertiary structures, and 3D models of the polypeptide (Källberg et al. 2012) and edited using Discovery Studio Visualization -BIOVIA (2017).

Subcellular localization of the sequence was assessed using three online software packages: PSORTb 3.0.2 (Yu et al. 2010), CELLO v.2.5 (Yu et al. 2004), and Gneg-mPLoc (Shen and Chou 2010). Consistent results in two out of three software predictions were considered. Signal peptides for cleavage were checked for using SignalP 4.1 (Petersen et al. 2011), and VaxiJen v2.0 was used for the prediction of protective antigens (Doytchinova and Flower 2007).

### Cloning, expression, and purification of polypeptide MLP15

Recombinant DNA sequences encoding rMLP15 were cloned in p*AE* expression vector (Ramos et al. 2004) using the restriction enzyme sites BamHI and HindIII. The resulting plasmid p*AE*/rMLP15 was used to transform *E. coli* BL21 Star™ (DE3) strain (Invitrogen, Carlsbad, CA, USA). Following verification of expression with a small-scale test, one colony was inoculated with 500 mL of lysogeny broth (LB, Bertani 1951). rMLP15 expression was induced by adding 1 M isopropyl β-d-1-thiogalactopyranoside (IPTG) to the culture (Sigma-Aldrich, St. Louis, MO, USA) for 3 h at 37 °C with agitation. Purification protocol was initiated with a brief sonication followed by centrifugation, in accordance with Simionatto et al. (2010). The supernatant containing the recombinant protein was solubilized in wash buffer (20 mM Tris–HCL, 8 M urea, 500 mM NaCl, 300 mM imidazole) at pH 8.0 (Simionatto et al. 2010). Affinity chromatography using HisTrap™ (GE Healthcare, Madison, WI, USA) 1 mL columns precharged with Ni-Sepharose was used to purify rMLP15. The concentration and purity of the purified rMLP15 were determined by the BCA Assay (Pierce, Rockford, IL, USA) and 12% sodium dodecyl sulfate–polyacrylamide gel electrophoresis (SDS–PAGE), respectively.

### Western blot with recombinant proteins

Western blot was performed with the purified rMLP15. After solubilizing in a sample buffer (62.5 mM Tris–HCl, 10% glycerol, 5% 2-β-mercaptoethanol, 2% SDS) at pH 6.8 and separated by 12% SDS–PAGE, proteins electroblotted onto nitrocellulose membranes (GE Healthcare, Madison, WI, USA) and incubated in 5% skim milk diluted in PBS-T (phosphate-buffered saline with Tween-20) overnight at 4 °C. The membranes were then washed thrice with PBS-T, and incubated for 1 h at 37 °C with an anti-6 × His-tag monoclonal antibody (Sigma-Aldrich, St. Louis, MO, USA) at a 1:6000 dilution in PBS-T. Following three PBS-T washes, the membranes were incubated with horseradish peroxidase (HRP)-conjugated mouse anti-IgG (Sigma-Aldrich, St. Louis, MO, USA), at 1:6000 and incubated for 1 h at 37 °C. DAB (3,3′-diaminobenzidine) was used for the visualization of reactive bands.

### Study population

Of the 24 patients with leprosy from whom sera were obtained for evaluation with ELISA, 10 were multibacillary (MB) and 14 paucibacillary (PB). The patients with positive bacilloscopy were classified as MB irrespective of the number of lesions (Reibel et al. 2015). Patients were clinically diagnosed by a dermatologist at a referral center for leprosy diagnosis and classified by bacilloscopy through a smear. The sera from 14 healthy individuals, was used as control. They were from a low endemic region with negative clinical diagnosis of leprosy and with no contact with leprosy and tuberculosis patients. All participating in the study were older than 18 years. The blood samples were collected by venipuncture and sera, once separated, were stored at − 20 °C until use. All participants in this study signed informed consent forms prior to enrolment and the study was approved by the Research Ethics Committee (1.816.093–11/09/2016) of the Federal University of Grande Dourados (UFGD).

### Enzyme-linked immunosorbent assay (ELISA)

The immunoreactivity of anti-rMLP15 IgG in patient sera was assessed using a modified ELISA protocol of Lima et al. (2017). In polystyrene 96-well ELISA plates (Kasvi™), 1 µg/well of rMLP15 in 100 μL of 0.1 M sodium carbonate buffer (pH 9.6) was coated and incubated for 16–18 h at 4 °C. Blocking was done with 200 µL/well of 5% skim milk in PBS-T for 2 h at 37 °C after washing the plates thrice with 0.05% PBS-T. Thereafter, the plates were washed three times with PBS-T and 100 µL/well of patient sera (diluted 1:50 in 5% skim milk in 0.05% PBS-T) was added. The plates were incubated at 37 °C and after three PBS-T washes, 100 μL/well of anti-human IgG peroxidase-conjugated antibody (Sigma-Aldrich, St. Louis, MO, USA) was added, diluted 1:10,000 in PBS-T, and further incubated at 37 °C for 1 h. The chromogenic reaction was developed by the addition of 100 μL/well of tetramethylbenzidine solution (Sigma-Aldrich, St. Louis, MO, USA) for 15 min at 37 °C, and the reaction was stopped with 100 μL/well of 2 N sulfuric acid. Absorbance at 450 nm was measured with a spectrophotometer (Bio-Rad, Hercules, CA, USA) and mean OD values were calculated from the serum samples assayed in triplicate.

### Statistical analysis

Statistical analyses included one-way analysis of variance (ANOVA), with Dunn’s test for multiple comparisons, and assessed using GraphPad Prism v.5.0 software (GraphPad Inc., La Jolla, CA, USA). The parameters of sensitivity, specificity, accuracy, and cutoff value based on Youden’s index were determined using a receiver operating characteristic (ROC) curve. Statistical significance was set at p < 0.05.

## Results

### Construction of polypeptide *rMLP15 and* in silico analysis

The epitopes selected from six *M. leprae* virulent proteins for rMLP15 construction are presented in Table 1.

**Table 1:** Virulence-associated epitopes selected for the construction of rMLP15

	Epitope	Protein	Accession	Start-MLP15
HLA-I	ALDTFGIPV	ML1358	NP_301968.1_64_92	169
	KLMGALDTF	ML1358	NP_301968.1_64_92	157
	IPASVSAPA	ML2055	NP_302372.1_257_287	13
	APIPASVSA	ML2055	NP_302372.1_257_287	61
	RAAVVQAAL	ML0885	NP_3t01670.1_245_270	25
	QMLEASSSV	ML1811	NP_302232.1_209_232	37
	SMDAAVAAL	ML1812	NP_302233.1_181_201	181
	RPVPVSTAR	ML1214	NP_301879.1_173_212	49
HLA-II	LRADSVLAV	ML1358	NP_301968.1_192_213	121
	ISLATVLSA	ML1358	NP_301968.1_158_181	145
	VVRDLRLA	ML1358	NP_301968.1_192_213	130
	WAILAIAVV	ML2055	NP_302372.1_1_78	73
	LAIAVVASA	ML2055	NP_302372.1_1_78	85
	ILAIAVVAS	ML2055	NP_302372.1_1_78	97
	VRPVPVSTA	ML1214	NP_301879.1_173_212	109

A 569 bp DNA sequence for the proposed rMLP15 was designed and translated into a 189 amino-acid polypeptide with a molecular weight of 17.3 kDa (Table 2). A BLASTp search for homologous proteins with *Mycobacterium* genera did not yield any results, indicating minimal cross-reactivity with other pathogens of the same genus. rMLP15 was not predicted to have premature cleavage or inadequate expression based on SignalP analysis, while predictions by the software packages yielded three possible intracellular localizations possible for rMLP15 (Table 2). The analysis by VaxiJen showed the possible use of rMLP15 in vaccine trials due to positivity for protective antigen (Table 2). A high pI of 10.5 and grand average of hydropathy (GRAVY index) score of 0.70 were found for rMLP15 using the ProtParam tool (Table 2).

**Table 2:** Physicochemical and functional properties of MLP15

Physicochemical property	
Number of amino acids	189
Molecular weight (Da)	17,310
Theoretical pI	10.5
Total of negatively charged residues (Asp + Glu)	7
Total of positively charged residues (Arg + Lys)	9
Extinction coefficient	5500
Abs. 0.1% (= 1 g/L)	0.318
Grand average of hydropathicity	0.709
Subcellular localization (psortb)	Cytoplasmic membrane
Subcellular localization (cello)	Extracellular
Subcellular localization (Gneg-mPLoc)	Cytoplasm
Peptide signal (SignalP)	No
Prediction of protective antigen (VaxiJen)	Probable antigen

Physicochemical parameters calculated by ProtParam, subcellular localization, signal peptide prediction and protective antigen prediction of MLP15. The software used is given in parentheses.

**Fig. 1:**
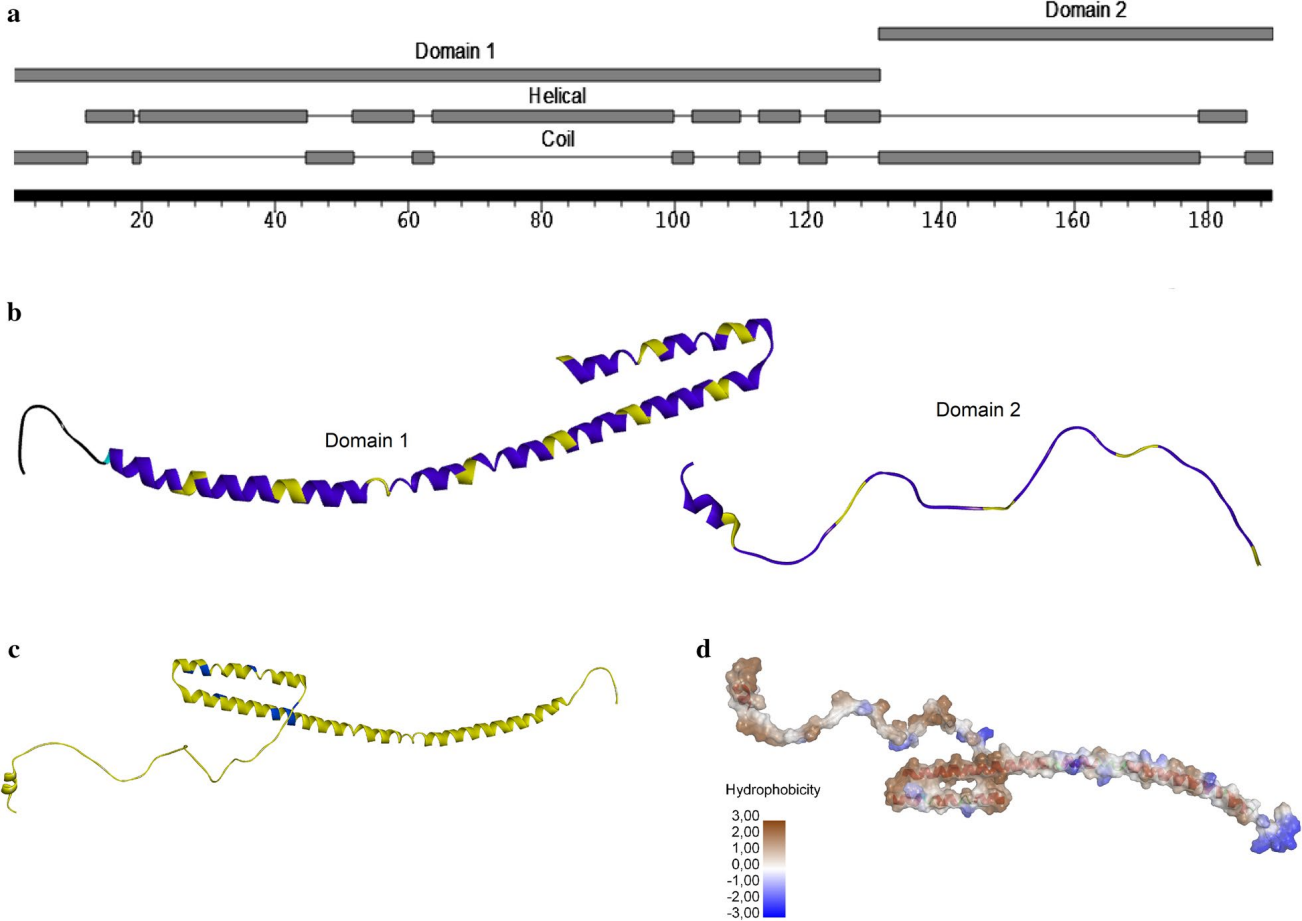
Primary structure and 3D rendering of MLP15. 3D rendering of MLP15. **a** The secondary structure of MLP15 indicating the location of the domains and turns and helices. **b** Tertiary extrusion prediction in the 3D model presenting the two main domains of MLP15. Blue: epitopes; and yellow: 3-Gly Linker. **c** Exposed surface of MLP15. Yellow: exposed amino acids; and blue: buried amino acids. **d** Hydrophobic surface of polypeptide MLP15 with color representation. Brown: more hydrophobic; blue: more hydrophilic; white: neutral. The analyses were done by RaptorX and edited in Discovery Studio Visualization-Biovia

The results of primary, secondary, and tertiary structure analyses performed using RaptorX are presented in Fig. 1. The protein structure appears to contain two domains of amino acids 1–130 (domain 1) and 131–189 (domain 2) (Fig. 1a and b). The protein showed high accessibility of residues with 132 exposed amino acids and only 8 buried, besides 49 amino acids with medium exposure (Fig. 1c). rMLP15 shows a primary coil and turn conformation, and achieves structural extension and linearization owing to the glycine linkers and the majority of exposed amino acids (Fig. 1a–c). The analysis also reveals rMLP15 to be essentially hydrophobic, with 89 hydrophobic, 76 neutral, and 24 hydrophilic amino acid residues (Fig. 1d).

### Cloning, expression, and purification

**Fig. 2:**
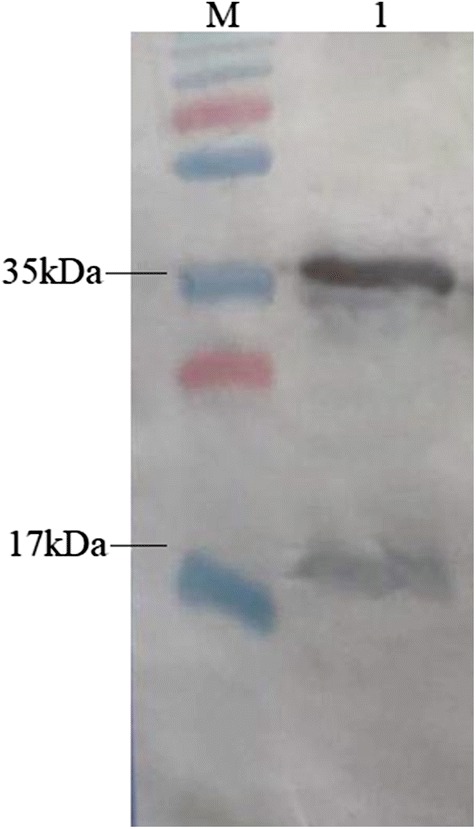
MLP15 recognized by ant-histidine in the western blot. Western blot of purified rMLP15 showing reaction with the bands of approximately 17 and 35 kDa. The protein was recognized by monoclonal antibody anti-6 His tag. *M* PageRuler Ladder (Thermo Scientific) and *1* purified His-rMLP15

The DNA sequence of MLP15 was cloned in a pAE expression vector and confirmed by restriction enzyme digestion, PCR, and sequencing. The highest level of MLP15 expression was obtained with the *E. coli* BL21 STAR (DE3) strain (Fig. 2). The protein yield obtained after purification was approximately 2.2 mg/L. After the purification, the presence of homodimers with approximately 35 kDa and monomers of 17 kDa (the predictable size of the rMPL15) were observed in western blot, using the anti-6 His tag (Fig. 2).

### ELISA-rMLP15

**Fig. 3:**
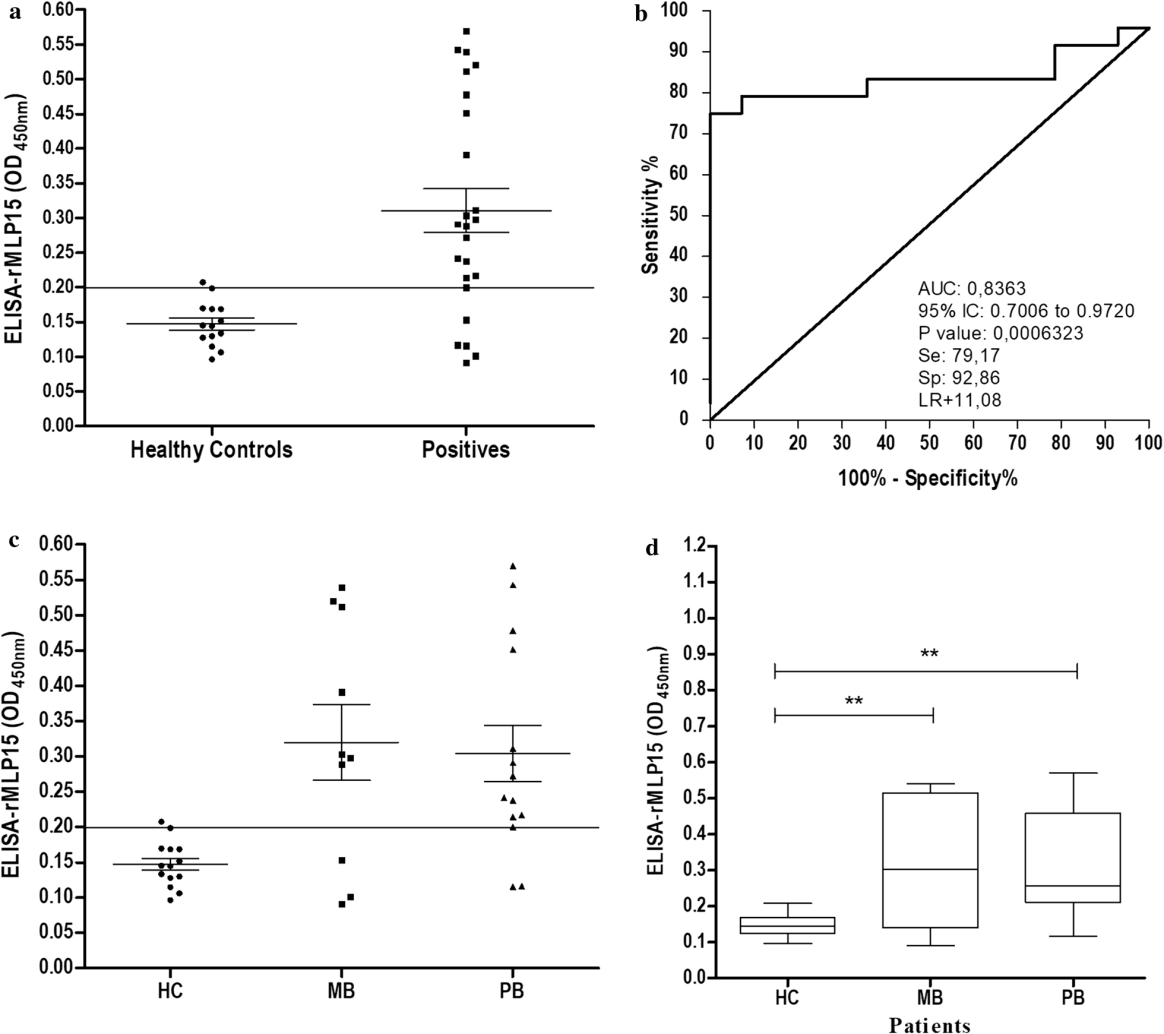
ELISA test performed with the sera from patients positive for leprosy using rMLP15. The results from indirect ELISA-rMLP15 for patient sera. **a** ELISA-rMLP15 in the sera from 24 leprosy patients and 14 healthy controls (HC). **b** Receiver operating characteristic (ROC) curve used to distinguish between positive and healthy controls (HC). **c** The levels of anti-rMLP15 IgG stratified paucibacillary patients (PB) and multibacillary patients (MB). **d** The levels of IgG anti-rMLP15 compared in HC (14), MB (10), and PB (14) patients; the serum levels were analyzed by ANOVA, followed by post hoc using Dunn’s test. **p < 0.001. *AUC* area under the curve, *CI* confidence interval, *Se* sensitivity, *Sp* specificity, *LR* likelihood ratio, *PB* paucibacillary, and *MB* multibacillary

The ELISA assays known for their proven diagnostic properties in leprosy were used to evaluate the performance of the recombinant antigen rMLP15. A ROC curve was constructed for the 24 patients and 14 HCs (Fig. 3), the cutoff value was based on the highest likelihood ratio (11.08) and the Youden index *J* was calculated (0.7203). rMLP15 showed an antibody response in 19 of 24 patients (79.17%) and 1 of 14 HCs (7.14%) from the sera evaluated in ELISA (Fig. 3a), thereby yielding a specificity rate of 92.86% (95% CI 66.13% to 99.82%), a sensitivity rate of 79.17% (95% CI 57.85% to 92.87%), and an area under the ROC curve (AUC) of 0.83 (95% CI 0.7006 to 0.9720) (Fig. 3b). In patients with leprosy, IgG levels were significantly elevated than in the control group (p < 0.001). Among patients with leprosy, rMLP15 was able to accurately identify 7 of 10 patients (70%) with MB and 12 of 14 PB patients (85.7%) (Fig. 3c) with a false positive rate of 7.14% (1 of 14 HCs). Statistical analysis did not reveal significant differences between the MB and PB groups, but both were significantly (p < 0.001) different from the HC group (Fig. 3d).

**Fig. 4:**
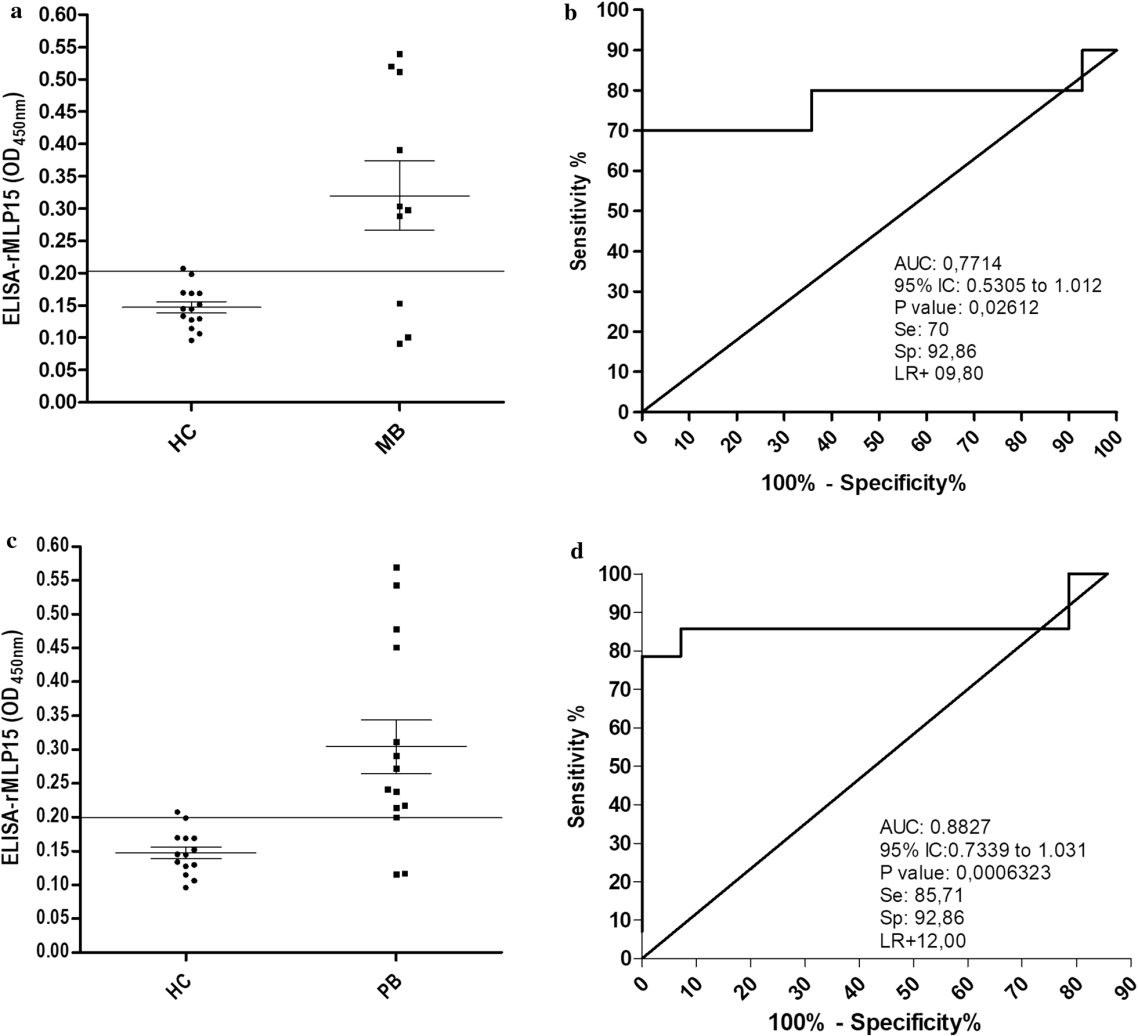
ELISA test stratifying MB and PB patients using rMLP15. The results of indirect ELISA-rMLP15 for MB and PB patient sera. **a** ELISA-rMLP15 in sera from 10 leprosy MB patients and 14 HC. **b** Receiver operating characteristic (ROC) curve used to distinguish between positive MB and HC. **c** ELISA-MLP15 in sera from 14 leprosy PB patients and 14 HC. **d** Receiver operating characteristic (ROC) curve used to distinguish between positive PB and HC. *AUC* area under the curve, *CI* confidence interval, *Se* sensitivity, *Sp* specificity, *LR* likelihood ratio, *HC* healthy control, *PB* paucibacillary, and *MB* multibacillary

ROC analysis was also performed on the subgroups of MB and PB patients (Fig. 4) and revealed a small increase in the cutoff values for MB patients, from 0.1993, in the overall analysis, to 0.2036 (Fig. 4a), while there was no change for PB data (Fig. 4c). Specificity was also unchanged for MB and PB subgroups in this analysis (92.86%). PB data showed a higher AUC value of 0.8827 (95% CI 0.7339 to 1.031) (Fig. 4d) than MB, 0.7714 (95% CI 0.5305 to 1.012) (Fig. 4b). The Youden index *J* was 0.6286 for MB and 0.7857 for PB.

## Discussion

The transmission of leprosy may be reduced by the use of tools that can accurately detect the disease at an early stage. Genomic sequencing has improved outcomes in this context by providing a more rational approach in the search of diagnostic tools for the diagnosis of many infectious diseases (Nagai et al. 2009). It has been established that the combined detection of humoral and cellular markers is efficient in diagnosing MB and PB leprosy patients (van Hooij et al. 2017, 2018). This study proposes a new design approach and a novel recombinant polypeptide for the serological detection of leprosy. A recombinant fusion protein based on six virulent *M. leprae* proteins (designated rMLP15), with established T-cell reactivity in leprosy patients, was used in an indirect ELISA to evaluate humoral responses in sera from leprosy-positive patients and healthy controls. Among the proteins that had epitopes selected for the construction of MLP15, ML2055 (5 epitopes) and ML1358 (5 epitopes), constituting 10/15 of MLP15, have been described as proteins with T cell and B cell epitopes that are immune reactive in the context of disease (Sampaio et al. 2011; Deval et al. 2016). rMLP15 demonstrated IgG reactivity in human sera for both MB and PB leprosy patients. These data confirm a plausible methodology that may be used to develop and improve new diagnostic tools for leprosy.

A number of bioinformatics analyses were carried out during the development process for rMLP15 to effectively guide the process and rationalize parameters to aid in better expression and purification of rMLP15. rMLP15 has been designed with some characteristics to optimize its use in diagnostics, like an extended chain structure with exposed amino acid residues to increase the possibility of recognition by specific immunoglobulins (Bergamaschi et al. 2019). The favored conformation of rMLP15 tertiary structure may be due to the ease of epitope–epitope interactions achieved by the presence of the 3-glycine linkers separating linear epitopes. Despite these optimizations, dimerization of the expressed rMLP15 was observed (Fig. 2), which may be due to intermolecular disulfide bond formation within homodimers or heterodimers and may result in higher oligomer formation (Futami et al. 2016). This could be explained by the high pI value (10.5) of rMLP15. It should be noted that this conformational observation does not seem to be a limiting factor in reactive antibody detection in leprosy patients.

The clinical course of disease progression in leprosy depends on the individual immune system (Alves et al. 2019). Genetic predisposition has been implicated to play a role in both disease susceptibility and host immune responses (Shankarkumar et al. 2003). The use of high-affinity HLA-binding linear epitopes in the construction of rMLP15 may aid in detecting both MB and PB leprosy patients. Available commercial tests like PGL-I or LID-1 protein-based tests can detect MB patients with high bacillary loads, but fail to detect PB leprosy patients, delegating them to a supportive test for treatment direction but not useful to detect early stages of the disease (Spencer et al. 2011; Geluk et al. 2011; van Hooij et al. 2017; Duthie et al. 2007; Leturiondo et al. 2019). PB leprosy, unlike MB leprosy, displays a dominant cellular phenotype with restricted anti-*M. leprae* antibody production. Our results show that rMLP15 was able to demonstrate seroreactivity in both PB and MB patients without significant differences in the levels of IgG antibodies between them.

rMLP15 demonstrated a higher detection rate of 79.17% (19 of 24) than by the NDO-LID^®^ test (62.8%, Orange Life, Rio de Janeiro, Brazil) (Frade et al. 2017). The detection rates among available commercial tests vary widely depending on patient populations, as well as disease spectrum, with lower rates in PB leprosy patients (van Hooij et al. 2017). The detection rates found in a study that used proteins like NDO-LID-1 and PGL-1 were 34% and 32% in PB patients versus 73.6% and 81% in MB individuals, respectively (Leturiondo et al. 2019). Although this test was able to exhibit better detection capabilities than standard tests using PGL-I and LID-I, monitoring of individuals in the early stages of the disease and/or PB patients remains a challenge (Frade et al. 2017).

This is the first study, to the best of our knowledge, describing enhanced serological detection rates for PB patient cases. rMLP15 detected 85.71% (12 of 14) PB patients and 70% (7 of 10) of MB patients, both with a specificity of 92.86% (Fig. 4). Additionally, the false positive rate with rMLP15 was 7.14% (1 of 14), which is lower than that reported in the PGL-I based tests (> 10%) (Alban et al. 2014). Although reported from a limited number of samples, these results encourage us to propose this peptide as a potential tool for early detection of leprosy, especially in cases with undetectable bacterial loads and few clinical signs, like in the PB patients and household contacts, that have a 4 to 9 times greater risk of developing leprosy than the general population (van Beers et al. 1999). Early detection is important not only to facilitate diagnosis and classification but also for decision-making regarding the candidates for prophylactic interventions, a key strategy for disrupting the transmission of disease (van Hooij et al. 2019).

The detection approach followed in this study shows that rMLP15 is able to diagnose cases of leprosy with high sensitivity and specificity multibacillary and paucibacillary patients. Our results put forward rMLP15 as a potential tool for leprosy diagnosis and the availability of such recombinant polypeptides could simplify future diagnostic test development. Further studies are needed to better characterize rMLP15 including testing with patient samples from different endemic regions, as well as in sera collected from individuals coming in contact with these patients and evaluate their ability to induce cellular responses.

## Data Availability

Not applicable
